# Oxygen as an Antidote to Asphyxia from Chloroform and Ether

**Published:** 1861-09

**Authors:** 


Oxygen as an Antidote to Asphyxia from Chloroform and Ether.—M. Ozanam has been experimenting with oxygen as an antidote to the asphyxia produced by anæsthetics, and finds that it acts much more promptly than atmospheric air to restore consciousness, producing its effects in less than half the time required by the latter. So long as there was the least sign of respiration, although the beatings of the heart had become imperceptible, consciousness was easily restored; in one instance, where both had ceased, it was powerless. These experiments confirm those of M. Duroy, made several years since. As a matter of precaution, then, it seems advisable for surgeons who employ chloroform, when undertaking important operations, to have at hand a quantity of oxygen gas ready for any emergency that may arise. Man resists the influence of chloroform better than the feeble animals which were experimented on; and so long as respiration continues, however slight or infrequent, oxygen will be likely to be efficient as a restorative.—*Boston Medical and Surgical Journal.*

